# Incidence of Tooth Loss in Adults: A 4-Year Population-Based Prospective Cohort Study

**DOI:** 10.1155/2017/6074703

**Published:** 2017-07-12

**Authors:** Manoelito Ferreira Silva-Junior, Marília Jesus Batista, Maria da Luz Rosário de Sousa

**Affiliations:** ^1^Department of Community Dentistry, Piracicaba Dental School, University of Campinas, Avenue Limeira 901, 13414-903 Piracicaba, SP, Brazil; ^2^Department of Community Health, Faculty of Medicine Jundiaí, R. Francisco Telles, No. 250, Vila Arens II, 13202-550 Jundiaí, SP, Brazil

## Abstract

**Objective:**

To verify the incidence of tooth loss in extended age group of adults in 4 years.

**Materials and Methods:**

The prospective cohort study assessed adults (20–64 years old) between 2011 and 2015, from Piracicaba, São Paulo, Brazil. The dependent variable was cumulative incidence of tooth loss, assessed by difference between missing teeth (M) of decayed, missing, and filled tooth index (DMFT) in 2011 and 2015. Participants were stratified into young (20–44 years old) and older (45–64 years old) adults. Mann–Whitney* U* test (*p* < 0.05) was used to compare the means of incidence of tooth loss between age groups.

**Results:**

After four years, 57.7% (*n* = 143) of adults were followed up and the mean incidence of tooth loss was 0.91 (SD = 1.65); among these, 51 adults (35.7%) who lost their teeth showed mean tooth loss of 2.55 (SD = 1.86). In older adults, incidence of tooth loss was higher (*p* = 0.008), but no difference between age groups was found when only adults with incidence of tooth loss were assessed (*p* = 0.844).

**Conclusion:**

There was higher incidence of tooth loss in older adults after four years, however, without difference between age groups when only those who lost teeth were evaluated.

## 1. Introduction

Tooth loss, still ranked among the hundred health conditions that most affect the world's population [1], is an oral condition that leads to functional, aesthetic, and social damage with impact on people's quality of life [2, 3] and is responsible for causing 7.6 million DALY (disability-adjusted life years) [1].

In spite of the more conservative philosophy within professional dental practice, where tooth extraction is treated as the last treatment option, there are cases in which this is the only choice [4, 5]. This is because time is a determinant factor in the progression and severity of oral diseases, such as caries and periodontal disease, and due to its cohort effect, the incidence of tooth loss during adulthood is higher [6, 7].

A review of 15 longitudinal studies from seven countries regarding tooth loss showed an annual incidence of the loss of one or more teeth ranging from 1.3% to 13.7% and the number of teeth lost varied from 3 to 38 per 100 subjects/year [8]. Although tooth loss can be prevented, its incidence has not declined in recent decades [1, 6] and it is still considered a public health care issue [1, 6, 9, 10]. In Brazil, the mean number of teeth lost in adults (35–44 years) is almost four times higher than that in adolescents (15–19 years) and half of mean number in older persons (65–74 years) [11]; therefore, it is important to investigate the distribution of tooth loss in the age range between the age groups of adolescents, adults, and older persons. This justified the use of an extended age group of adults in the present study.

The clinical aspects of tooth loss, such as most affected teeth, their distribution [12, 13], and condition before tooth extraction, have been more exploited in clinical studies [4, 14, 15] and may not correspond to the reality of the population. Observational studies with adults are rarely found in the literature and may present more detailed data on clinical conditions of tooth loss [16]. Tooth loss studies are usually cross-sectional [9, 11, 12, 14, 17–20] and assess factors associated with this condition, mainly socioeconomic and oral health service utilization [8–12, 14, 15, 17–20].

The data on the distribution of tooth loss in a population-based cohort, mainly in an extended age group of adults, would be able to infer more reliable data for the planning of actions in public health and may also serve as a basis for verifying the impact of the public health policies implemented. To this end, the objective of this study was to verify the incidence of tooth loss in an extended age group of adults (20–64 years) in a period of 4 years.

## 2. Material and Methods

### 2.1. Study Design and Location

This prospective cohort study conducted in Piracicaba, São Paulo, Brazil, was part of a dissertation entitled ‘‘Longitudinal tooth loss study in adults and associated factors” [5].

### 2.2. Ethical Aspects

This research was approved by the Research Ethics Committee of the Piracicaba Dental School (CEP-FOP/Unicamp) (177/2009).

### 2.3. Population and Sample

#### 2.3.1. Baseline

To calculate the representative sample of adults (20–64 years) living in Piracicaba, São Paulo, oral health conditions were assessed in different age groups and two different calculations were estimated for the sample size of young adults (20–44 years) and older adults (45–64 years). We adopted a design effect of 1.5; margin of error of 10.0%; and 95.0% confidence interval, data concerning the prevalence of caries for each age group (70.2% and 90.9%, resp. [18]), and added 20% to the total to compensate occasional losses. The sample size for adults aged 20–44 years was 172, and for those aged 45–64 years, 68, totaling 240 adults. We added 30% to the final sample size for selecting adults, foreseeing the possibility of losses and refusals, resulting in 342 households, 11.4 households for each census tract [3].

The sample selection was planned based on the Brazilian Demographic Census (2000) [21], the latest data compiled at the time when the study was conducted. The Piracicaba population of adults from 20 to 64 years old was 202, 131; 30 census tracts were randomly selected using probability sampling; 11 households were randomly selected in each sector, according to a varying fraction determined by the number of households. One adult per house was examined [3].

#### 2.3.2. Follow-Up

For the purpose of following up the same subjects, the home census tracts related to their current residences were not considered [5].

### 2.4. Data Collection

#### 2.4.1. Baseline

Data collection took place between June and September 2011. The research consisted of one clinical oral examination and one interview. Clinical oral examinations were measured by the number of decayed, missing, and filled teeth index (DMFT) and need for dental caries treatment was performed in the households, under artificial lighting without prior prophylaxis or drying, using CPI-probes and front surface mouth mirrors, as recommended by the World Health Organization [22]. In addition, each volunteer answered a questionnaire on demographic (sex, age, race, and marital status) and socioeconomic (family income and education) factors.

Inclusion criteria were living in one of the residences drawn in Piracicaba, São Paulo, and to be between 20 and 64 years of age in 2011. Exclusion criteria were those with physical and psychological conditions that would interfere in the clinical procedures or in the understanding of the questionnaire [3].

At baseline, one examiner conducted the study, after being trained by a benchmark examiner, through theoretical and practical discussions, lasting for a total of 16 hours, obtaining agreement equal to or greater than 90.0% for coronal caries and treatment needs for dental caries. Intraexaminer agreement was from 96.5% to 100.0% and the Kappa coefficient ranged from 0.89 to 1.00, within reliability standards [3].

At this stage, there was a loss of 24.0% (*n* = 82) adults because they did not agree to participate in the study or were not found during one of the three visits; however, a minimum number of 240 adults was obtained, or representativeness of adults of the studied municipality. At baseline, the sample was composed of 248 adults, representing the 149,635 adults (20–64 years old) living in Piracicaba, São Paulo, Brazil.

#### 2.4.2. Follow-Up

Data collection was made between June and September 2015. Inclusion criterion was to have participated in the independent baseline of the current census tract of residences in 2015. Exclusion criterion was physical and psychological conditions that would interfere with the clinical procedures [5].

During follow-up, two examiners participated in this stage of data collection; they were trained by benchmark examiner (baseline examiner) with theoretical and practical discussions, calibrated in a total of 20 hours, and obtained at least 90.0% agreement relative to coronal caries and treatment needs for dental caries. Intraexaminer and interexaminer agreement was from 96.5% to 100.0% and the Kappa coefficient ranged from 0.89 to 1.00, within reliability standards [5].

The same individuals were sought at their addresses and invited to participate in the study. If the individual was not found, at least three more attempts were made. Participants signed the Term of Free and Informed Consent to participate in the study. The same oral clinical conditions were assessed, using the same criteria and examination protocol as that used at baseline [3]. At the time of data collection, each individual kept the same baseline identification.

At this stage, the sample was composed of 143 (follow-up rate = 57.7%) adults. The reasons for not participating were 64 (25.8%) could not be found, 33 (13.3%) refused to participate, and 8 (3.2%) died [5].

### 2.5. Variables

The dependent variable was cumulative incidence of tooth loss, assessed by the difference between missing teeth (M) of decayed, missing, and filled teeth index (DMFT) in 2011 and 2015. Missing teeth (M) were considered the teeth with codes 4 (tooth loss due to dental caries experience) and 5 (tooth loss due to other causes) of the DMFT index. Treatment needs for dental caries were determined as restorative (in one or more surfaces and a single crown) and endodontic treatment and extraction. For calculating the clinical variables, the 32 teeth were considered.

As reference for the sample characterization, we used the socioeconomic and demographic data collected at baseline. Age was stratified into two groups: young (20–44 years) and older (45–64 years) adults at baseline (2011), so that there would be no transition among the studied groups. Racial groups were defined by self-declaration, and these were categorized as white and non-white (black, brown, yellow, or indigenous). Marital status was categorized as stable relationship (married or cohabitation) and nonstable relationship (single, divorced, or widowed). Family income was categorized as low (less than 1 minimum wage (MW)), average (1-2 MW), and high (greater than 2 MW). Educational level was categorized according to the number of years of completed education (≤4 years, between 5–10 years, and ≥11 years).

### 2.6. Data Analysis

The data were tabulated with Statistical Package for the Social Sciences version 20.0 (SPSS) and Microsoft Excel®. A descriptive analysis was performed, thus obtaining the absolute and percentage distribution, mean, and standard deviation (SD) of the studied variables. The Mann–Whitney* U* test (*p* < 0.05) was used to compare the mean values of incidence of tooth loss between age groups.

## 3. Results

The majority of the participants were women (72.0%), older (51.0%), in stable relationship (78.3%), and with a mean family income (63.6%) and had studied for more than 11 years (53.1%) (Table 1).

After the 4-year follow-up, 12.6% (*n* = 18) adults still had no tooth loss, 1.4% (*n* = 2) presented their first tooth loss and one 0.7% (*n* = 1) adult became edentulous.

**Figure 1:**
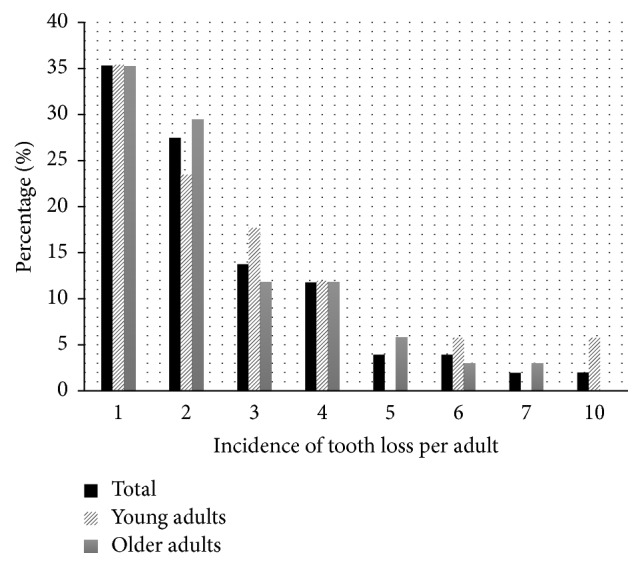
Frequency of total incidence and by age group of the number of teeth lost by adults in the 4-year follow-up. Piracicaba, São Paulo, Brazil, 2011–2015.

A total of 51 adults (35.7%) had lost at least one tooth, among these 24.3% (*n* = 17) of the young adults and 46.6% (*n* = 34) of the older adults. There was a higher incidence of 35.0% (*n* = 18) of one tooth loss per individual (Figure 1).

Table 2 shows the mean incidence of tooth loss was 0.91 (SD = 1.65); among these, 51 adults (35.7%) showed the loss of 2.55 (SD = 1.86) teeth. The incidence of tooth loss in older adults (*p* = 0.008) was higher, but there was no difference between age groups when only adults with incidence of tooth loss were assessed (*p* = 0.844) (Table 2).

**Figure 2:**
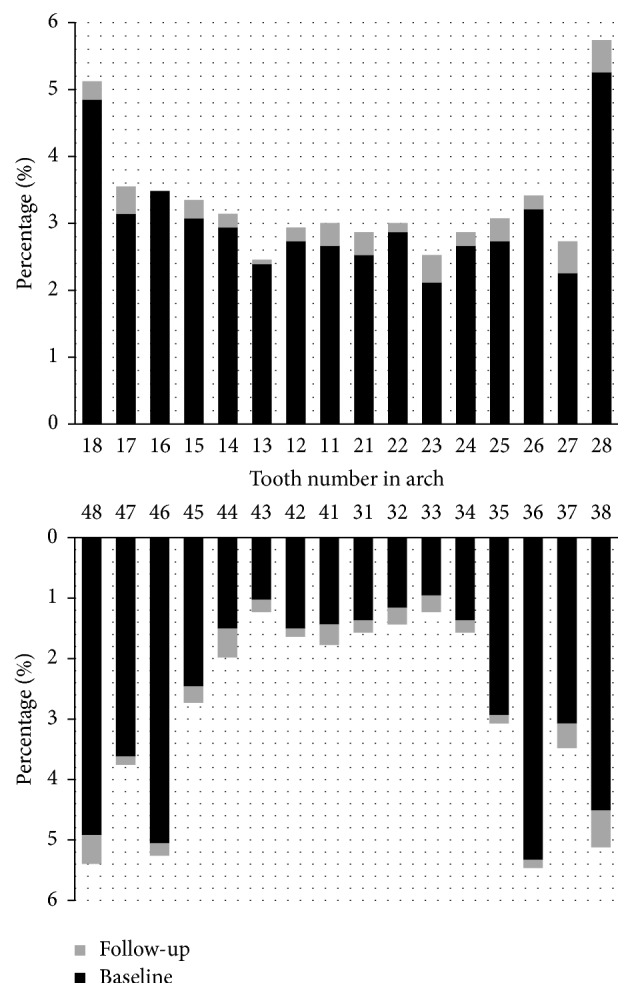
Percentage of tooth loss according to affected teeth in adults at the baseline and follow-up. Piracicaba, São Paulo, Brazil, 2011–2015.

At baseline, the prevalence of tooth loss was higher in the maxilla (53.7%), with the maxillary molars being the most affected (29.1%) and the front teeth the least affected (8.1%). In the follow-up, the incidence was similar in the maxilla (50.8%) and mandible (49.2%); the mandibular molars (22.3%) were the most affected teeth, and maxillary premolars (11.5%) the least affected (Figure 2).

For the adults who had incidence of tooth loss, in total 130 teeth were lost in the last four years; and at baseline, the majority had treatment needs for caries, which was restorative treatment (64.9%) (Table 3).

## 4. Discussion

The findings of this population-based prospective cohort study with a sample of adults in expanded age group are of great relevance for understanding tooth loss. While data were compiled on tooth loss and treatment needs for dental caries at baseline, in the follow-up it was possible to check clinical changes in the teeth over the course of time. Over one-third of the sample had incidence of tooth loss in 4 years, and the incidence increased according to the age group studied. The incidence of tooth loss among young adults was, however, equal to that of older adults, considering only individuals who had lost teeth.

The present study presented some limitations, such as the higher number of women participating, a fact also verified in other reports of home collection [3, 9, 11–13]. The sample loss expected in cohort studies also occurred in this study [8, 15, 16, 23]. However, the sample retained the characteristic of being mostly composed of women. Among the treatment needs identified relative to the lost teeth, in the present study, only caries requirements were considered; and other previous conditions of the teeth, such as previous restorations or periodontal conditions were not verified. Thus, the authors recommend that future cohort studies should include other oral health care needs with the purpose of preventing future tooth loss.

In this study, expanded age groups and stratification into the two age groups of our study allowed us to verify important differences between the distribution of tooth loss in young adults and older adults. In spite of differences in the methodology of longitudinal tooth loss studies, such as the exclusion of third molars from analysis [11, 12], use of a restricted age group, between 35 and 44 years [22], as recommended by WHO, and number of years of follow-up, it was possible for the authors of the present study to verify a methodological pattern of higher incidence of tooth loss as the period of the follow-up increased [15, 16, 20] and the age group studied was older [24]. Moreover, even if most studies considered age an associated or risk factor of tooth loss [5, 18, 19, 25], this association is questionable, because there is no established evidence between tooth loss and the physiology of aging [24].

In present study, the number of adults with incidence of tooth loss was almost twice as high among older adults. The mean number of teeth lost was higher in older adults when compared with the total sample, whereas there was no difference between the mean number of teeth lost when the age groups studied were compared between adults with incidence of tooth loss. This result denotes a polarization of tooth loss in young adults, as happens with dental caries in children, because the highest burden of the condition is concentrated in a small portion of individuals [26]. Although the incidence of tooth loss was restricted to a small number of young adults, it was equal to the mean incidence in older adults.

The highest incidence of one tooth lost per individual was in agreement with values in the literature [15, 16, 25]. In present study, only one individual became edentulous. In the past decades, studies have verified a reduction in the prevalence of edentulism [27], which may denote less invasive oral health care practices today, as tooth extraction is considered the last treatment option.

We also found that the incidence of tooth loss per group of teeth was similar to the data found for prevalence of tooth loss [18]. When prevalence of tooth loss at baseline and follow-up was assessed, distribution was similar to that found in the literature [15, 18]; the mandibular molars were the first teeth lost and the mandibular front teeth are those that remained longest. Examination of the incidence of tooth loss showed a different pattern: premolars and teeth in the mandibular dental arch were least frequently missing. This may be explained by the fact that this study was conducted with expanded age groups and included individuals in different stages of life, but it should be noted that the molars continued to be the most frequently missing teeth. Another aspect that could justify why the mandibular teeth are kept, mainly the mandibular anterior teeth, is the professional choice of these teeth as prosthetic abutments for greater retention of prosthesis in the mandibular ridge [5].

In the evaluation of each tooth that was lost at follow-up and the treatment needs for dental caries at baseline, the majority of participants needed restorative treatment. From this, we inferred a greater need for low complexity treatments that could have been made by primary oral health care in the initial stages of dental caries [17]. Most studies have, however, demonstrated the need for more dental services in secondary care for adults [9, 19], mostly due to the high accumulated demand [28]. This result becomes important since tooth loss is a direct consequence of lack of dental caries treatment. Therefore, it is difficult to think of reducing tooth loss without thinking of reducing tooth decay. This restates the necessity of providing proper knowledge on oral hygiene and diet, for example, rational consumption of sugar, regular use of the dental services, and healthy choices to maintain and keep teeth functional throughout their entire life time.

From this perspective, to meet the oral health care needs of the Brazilian people and to achieve integrality in health care, oral health care was included in the Unified Health System (SUS), with the creation of the National Oral Health Policy in 2003 and deployment of Dental Specialties Centers (DSC). DSC provides users with specialized oral health treatments capable of rehabilitating teeth with severe damage and tissue loss [9].

Although Brazil has developed oral health policies, for example, increase in the number of Oral Health Teams (OHT) in the Family Health Strategy, other aspects should be discussed. As verified in our study, although large investments have been made to expand OHT, in Piracicaba as well, where 14-OHT modality type I, and two DSC have been implemented up to now, this is still not enough to reduce dental caries and its main threat to the adult population: tooth loss. Nevertheless, in Brazil these changes and investments in public oral health are usually made over time; therefore, longitudinal studies are necessary to measure these aspects.

Outcomes from this study may assist with planning adult oral health policies, because they demonstrated the eminent need for promoting basic oral health care. This restates the necessity to focus on oral health care promotion regardless of the age group of adults studied, as a continuous approach, and as early as possible, in order to prevent oral health diseases and their worsening, because they may have an impact on the other life cycles of individuals, and lead to tooth loss.

Access of the economically active population, the greater part of the adult population, to health care services becomes difficult where opening hours are concerned. Alternative opening hours, at night or weekends, would facilitate access to public dental services. Another important aspect of the discussion on tooth loss is related to professional practice, often still centered on the biomedical model, strengthened by repetitive restorative cycles and without prioritizing risk factors, preventing oral health diseases, and promoting oral health care. Further aspects concern personal motivation, both relative to the late demand for treatment, that is, motivated by pain or beliefs that make individuals choose tooth extraction [5, 26], and the low value they assign to their teeth. These hypotheses, however, require further research, in order to provide more subjective explanations of factors that cause tooth loss in the long-term.

## 5. Conclusion

After four years, it was possible to verify a higher incidence of tooth loss in older adults, however, without difference between age groups when only those who lost teeth were evaluated.

## Figures and Tables

**Table 1:** Demographic and socioeconomic characteristics of sample of adults living in Piracicaba, São Paulo, Brazil.

Characteristics	*n* (%)
Demographics	
*Sex*	
Male	40 (28.0)
Female	103 (72.0)
*Age*	
Young adults	70 (49.0)
Older adults	73 (51.0)
*Race*	
White	122 (85.3)
No white	21 (14.7)
*Marital status*	
Stable relationship	112 (78.3)
No stable relationship	31 (21.7)
Socioeconomics	
*Family income*	
Low	23 (16.1)
Average	91 (63.6)
High	25 (17.5)
Missing	4 (2.8)
*Education*	
≤4 years	30 (21.0)
5–10 years	37 (25.9)
≥11 years	76 (53.1)

*Note*. ^*∗*^Reference data (2011).

**Table 2:** Number of teeth lost by age group of adults for simple total and adults with incidence of tooth loss in 4 years of follow-up. Piracicaba, São Paulo, Brazil, 2011–2015.

Incidence of tooth loss	Total	Young adults	Older adults	*p* value^*∗*^
	Mean (SD)	Mean (SD)	Mean (SD)	
Simple total (*n* = 143)	0.91 (1.65)	0.67 (1.64)	1.14 (1.64)	0.008
Among adults with incidence (*n* = 51)	2.55 (1.86)	2.76 (2.33)	2.44 (1.60)	0.844

^*∗*^Mann–Whitney *U* test (*p* < 0.05).

**Table 3:** Absolute frequency of treatment needs for dental caries at baseline (2011) and incidence of tooth loss (2015) according to the number of teeth in the arch. Piracicaba, São Paulo, Brazil, 2011–2015.

Tooth number in arch	Treatment needs for dental caries (2011)				Incidence of tooth loss (2011–2015)
	Restorative *n*	Endodontics *n*	Extraction *n*	Total *n*	Total *n*
18	4	0	3	7	4
17	1	2	2	5	6
16	4	0	0	4	0
15	3	1	1	5	4
14	1	1	1	3	3
13	3	0	2	5	1
12	2	0	0	2	3
11	6	0	0	6	5
21	7	0	0	7	5
22	2	2	0	4	2
23	4	0	1	5	6
24	1	2	0	3	3
25	2	1	0	3	5
26	3	4	0	7	3
27	2	0	2	4	7
28	1	0	0	1	7
38	4	0	2	6	9
37	1	3	1	5	6
36	6	2	1	9	2
35	1	0	0	1	2
34	1	0	0	1	3
33	3	0	0	3	4
32	2	0	0	2	4
31	1	0	0	1	3
41	2	0	0	2	5
42	3	0	0	3	2
43	1	0	0	1	3
44	4	2	1	7	7
45	2	1	0	3	4
46	3	2	1	6	3
47	3	1	0	4	2
48	2	1	3	6	7

Total	85 (64.9%)	25 (19.1%)	21 (16.0%)	131 (100.0)	130 (100.0)
